# Concerted evolution of body mass and cell size: similar patterns among species of birds (Galliformes) and mammals (Rodentia)

**DOI:** 10.1242/bio.029603

**Published:** 2018-03-14

**Authors:** Marcin Czarnoleski, Anna Maria Labecka, Dominika Dragosz-Kluska, Tomasz Pis, Katarzyna Pawlik, Filip Kapustka, Wincenty M. Kilarski, Jan Kozłowski

**Affiliations:** 1 Institute of Environmental Sciences, Jagiellonian University, Gronostajowa 7, 30-387 Kraków, Poland; 2 Institute of Zoology, Department of Biology and Cell Imaging, Jagiellonian University, Gronostajowa 9, 30-387 Kraków, Poland

**Keywords:** Allometry, BMR, Body size, Concerted evolution, Interspecific scaling, Karyoplasmic ratio, Metabolic rate, Optimal cell size, Species diversity

## Abstract

Cell size plays a role in body size evolution and environmental adaptations. Addressing these roles, we studied body mass and cell size in Galliformes birds and Rodentia mammals, and collected published data on their genome sizes. In birds, we measured erythrocyte nuclei and basal metabolic rates (BMRs). In birds and mammals, larger species consistently evolved larger cells for five cell types (erythrocytes, enterocytes, chondrocytes, skin epithelial cells, and kidney proximal tubule cells) and evolved smaller hepatocytes. We found no evidence that cell size differences originated through genome size changes. We conclude that the organism-wide coordination of cell size changes might be an evolutionarily conservative characteristic, and the convergent evolutionary body size and cell size changes in Galliformes and Rodentia suggest the adaptive significance of cell size. Recent theory predicts that species evolving larger cells waste less energy on tissue maintenance but have reduced capacities to deliver oxygen to mitochondria and metabolize resources. Indeed, birds with larger size of the abovementioned cell types and smaller hepatocytes have evolved lower mass-specific BMRs. We propose that the inconsistent pattern in hepatocytes derives from the efficient delivery system to hepatocytes, combined with their intense involvement in supracellular function and anabolic activity.

## Abstract (precis)

**Summary:** In Galliformes birds and Rodentia mammals, larger species evolved larger cells of different cell types; in Galliformes birds, large-cell species evolved lower metabolic rates per unit mass.

## INTRODUCTION

Whether they are bacteria, protists, fungi, plants or animals, living things have evolved a plethora of different body plans and life strategies, resulting in dramatic differences in body mass among species. We know surprisingly little about the cellular mechanisms involved in the origin of this variance. The evolution of larger or smaller organisms can occur simply through changes in cell number, which should help preserve the fundamental physiological characteristics of single cells in a body. However, a change in cell number may affect physiological performance if the number of cells in an organ affects organ function or if tissue maintenance depends on cell number and cell size. According to the theory of optimal cell size (TOCS) (Atkinson et al., 2006; Czarnoleski et al., 2015a, 2016; Davison, 1956; Kozłowski et al., 2003; Szarski, 1983), cell size is optimized according to the organism's requirements, and its adaptive value depends on the cost associated with the maintenance of the cell membrane and the capacity of the cell to perform physiological functions. To maintain the functioning of cell membranes, an organism devotes substantial amounts of energy to the maintenance of the physical properties of cell membranes (Engl and Attwell, 2015) and to the generation of electro-chemical potentials across their surface (Rolfe and Brown, 1997). All else being equal, energetic demand per unit mass should be lower in larger organisms if body mass evolves in concert with cell size. A larger body that consists of not only more but also larger cells has a smaller amount of cell membranes per unit of tissue mass, which should lower its metabolic costs per unit of body mass. Nevertheless, organs with large cells are expected to metabolize at a slower rate than are organs with small cells because of the smaller surface area of cells available for the exchange of substrates and products, the longer distances involved in intracellular diffusion, and the fewer nuclei for transcription in organs with large cells (Czarnoleski et al., 2015b).

To date, the TOCS has been used to address the origin of cell size variance in ectotherms exposed to environmental gradients (Czarnoleski et al., 2015b; Walczyńska et al., 2015), and in ectotherms and endotherms characterized by different metabolic rates (Hermaniuk et al., 2017; Maciak et al., 2011, 2014; Starostová et al., 2013). Most of the studies on these topics have focused on one cell type and generalized their results to organism-wide trends in cell size, but sound conclusions regarding the cellular architecture of an organism require information about the cell sizes in different body parts and on the cell sizes originating from different germ layers. To address this problem, we studied the body mass and size of erythrocytes, chondrocytes, hepatocytes, enterocytes, epithelial skin cells and kidney cells in different species of Galliformes birds and Rodentia mammals. We also measured nuclei in the erythrocytes of birds, and used the database by Gregory (2017) to extract published data on the genome sizes of the studied species where available. We first aimed to examine whether species diverged with respect to cell size and whether this divergence involved coordinated changes in cell size in different tissue types (hypothesis I). Following Gregory (2001) and Kozłowski et al. (2003), we predicted that such evolution involves alterations in genome size. To explore this idea, we examined links between the sizes of cells, nuclei and genomes. We also compared the karyoplasmic (nucleus-to-cell size) ratios of erythrocytes among bird species. Following the evidence of Cavalier-Smith (2005), we expected no variance in this ratio. Next, we tested whether interspecific differences in body mass evolved in association with changes in cell size (hypothesis II) or exclusively through changes in the number of cells. Note that if hypothesis I holds, we might then expect that body mass evolved with an involvement of organism-wide changes in cell size. Finally, focusing on birds, we measured basal metabolic rates (BMRs) in the same individuals for which we collected information about cell size. In this way, we examined a prediction regarding the TOCS stating that the evolution of large-celled species should be associated with lower mass-specific costs of tissue maintenance (hypothesis III) (Kozłowski et al., 2003). Note that if hypothesis II holds, then following hypothesis III, BMRs should increase with body mass at a ratio of less than 1:1 (negative allometric scaling with mass), which would correspond to lower mass-specific BMRs in larger species.

## RESULTS

According to our principal component analysis (PCA) (Table 1), in both groups of animals, the sizes of erythrocytes, enterocytes, chondrocytes, epithelial skin cells and kidney cells loaded positively on the first principal component (PC1), and the size of hepatocytes loaded negatively on PC1. This pattern indicates that across species, cells in five tissues had positively correlated sizes, and the sizes of these cells were inversely related to the size of hepatocytes. The second principal component (PC2) mainly explained the positive effects of hepatocytes (mammals) or the positive effects of hepatocytes and erythrocytes and negative effects of duodenal enterocytes (birds). Data on the raw measures of cell size are provided in Table S1.

**Table 1.**
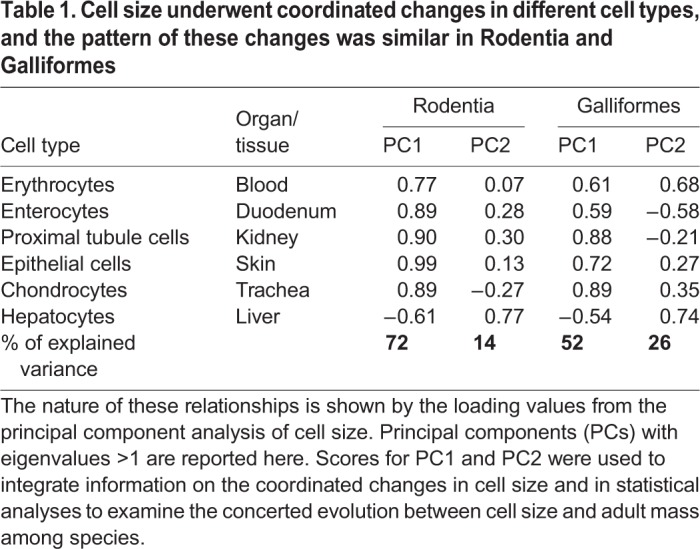
**Cell size underwent coordinated changes in different cell types, and the pattern of these changes was similar in Rodentia and Galliformes**

**Fig. 1.**
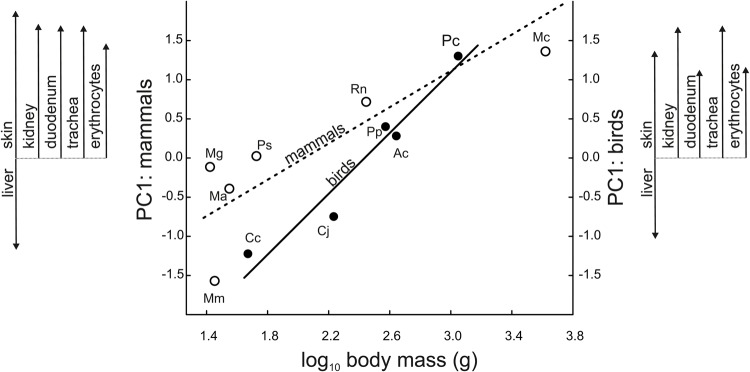
**In birds and mammals, larger species have evolved smaller hepatocytes but larger erythrocytes, chondrocytes, enterocytes, kidney cells and skin cells.** Lines represent the standardized major axis (mammals: y=−2.36+1.159x; birds: y=−4.72+1.940x). Symbols represent the species means calculated from the data for five individuals and are marked with initials for each species name. Mammals: Ma, *Microtus arvalis*; Mc, *Myocastor coypus*; Mg, *Myodes glareolus*; Mm, *Mus musculus*; Ps, *Phodopus sungorus*; Rn, *Rattus norvegicus*. Birds: Ac, *Alectoris chukar*; Cc, *Coturnix chinensis*; Cj, *Coturnix japonica*; Pc, *Phasianus colchicus*; Pp, *Perdix perdix*. PC1 is the first principal component in the principal component analysis of cell size. Scores for PC1 were used to integrate information on the coordinated changes in cell size. Arrows indicate the loading values for cell size in different organs/tissues from PC1 (see Table 1), demonstrating the nature of cell size relationships.

In total, the two principal components (PCs) explained 86% of the interspecific variance in cell size in mammals and 78% in birds. The major part of this variance, explained by PC1 (72% in mammals and 52% in birds), was related to interspecific differences in body mass (Fig. 1), as indicated by positive correlations between the PC1 scores and body mass in mammals (r=0.84, *P*=0.04) and birds (r=0.97, *P*=0.006). In other words, larger species have evolved smaller hepatocytes, but larger erythrocytes, chondrocytes, enterocytes, kidney cells and skin cells. Information on the correlation between body mass and raw cell size is provided in Table S2. The part of interspecific variance in cell size explained by PC2 (14% in mammals and 26% in birds) was unrelated to body mass ‒ PC2 scores did not correlate with body mass (mammals: r=0.14, *P*=0.79 and birds: r=0.02, *P*=0.98).

**Fig. 2.**
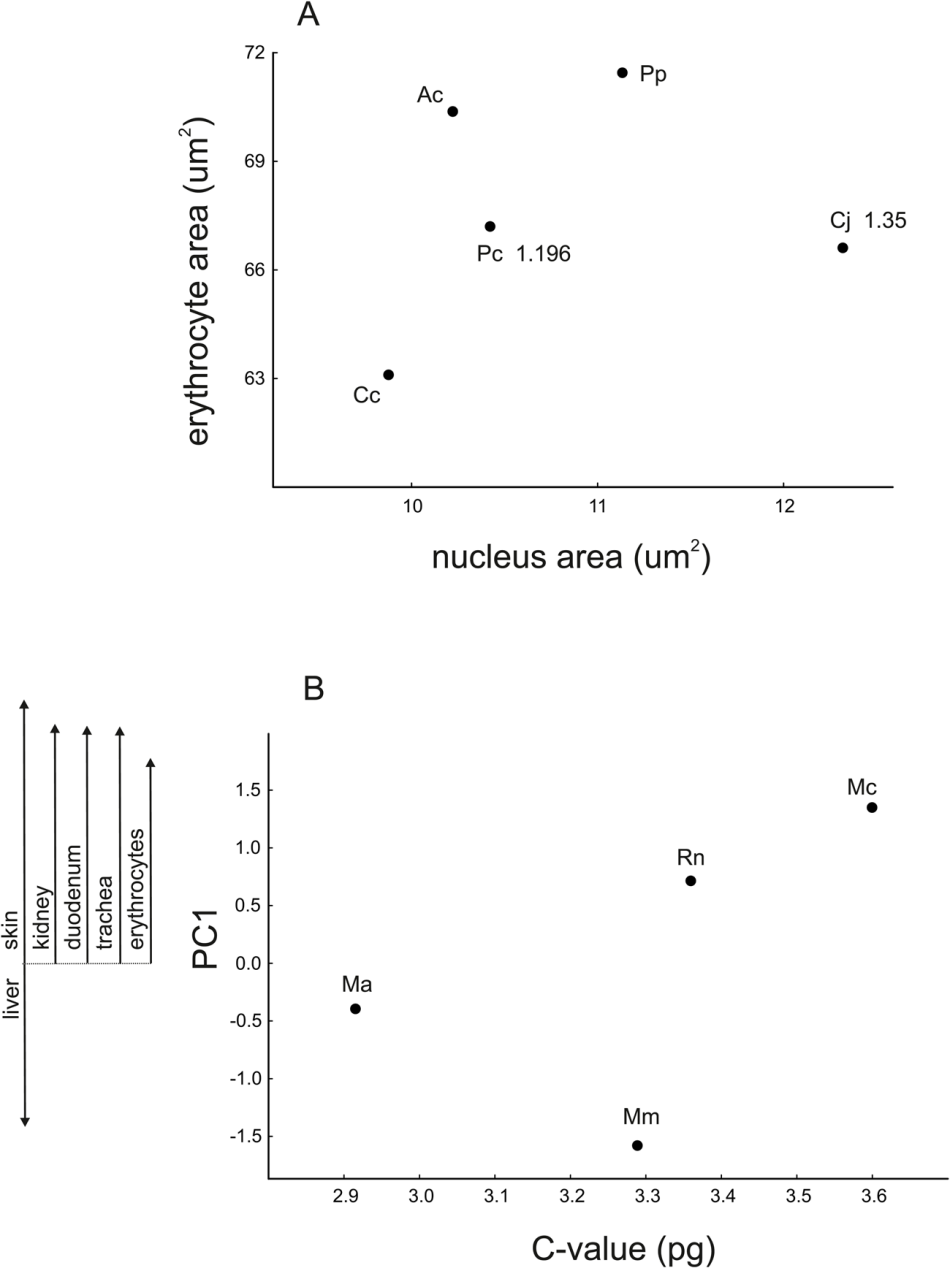
**In birds (A), the size of erythrocytes did not correlate with the size of erythrocyte nuclei, and in mammals (B), cell size did not correlate with genome size (C-value).** Data about genome size (C-value; pg) were available for only two bird species, and they are shown in A near the initials of the species. C-values were not available for two species of mammals. Symbols are species means, marked with the initials for each species name. Panel A: Ac, *Alectoris chukar*; Cc, *Coturnix chinensis*; Cj, *Coturnix japonica*; Pc, *Phasianus colchicus*; Pp, *Perdix perdix*. Panel B: Mm, *Mus musculus*; Mc, *Myocastor coypus*; Ma, *Microtus arvalis*; Rn, *Rattus norvegicus*. PC1 is the first principal component in the principal component analysis of cell size. Scores for PC1 were used to integrate information on the coordinated changes in cell size. Arrows indicate loading values for cell size in different organs/tissues from PC1 (see Table 1), demonstrating the nature of cell size relationships.

In birds, karyoplasmic ratios for erythrocytes differed significantly among species (*F*_4,20_=8.40, *P*=0.001). The mean sizes of erythrocytes and their nuclei were not correlated across species (r=0.20, *P*=0.75; Fig. 2A). In the two species of birds for which we obtained information about genome size, the species with the larger genome had slightly larger erythrocyte nuclei and smaller erythrocytes (Fig. 2A), although this difference is most likely not statistically significant. In the four species of mammals for which we had information on genome size, genome size was not significantly correlated with cell size for PC1 (r=0.57, *P*=0.43; Fig. 2B) or PC2 (r=0.12, *P*=0.92).

**Fig. 3.**
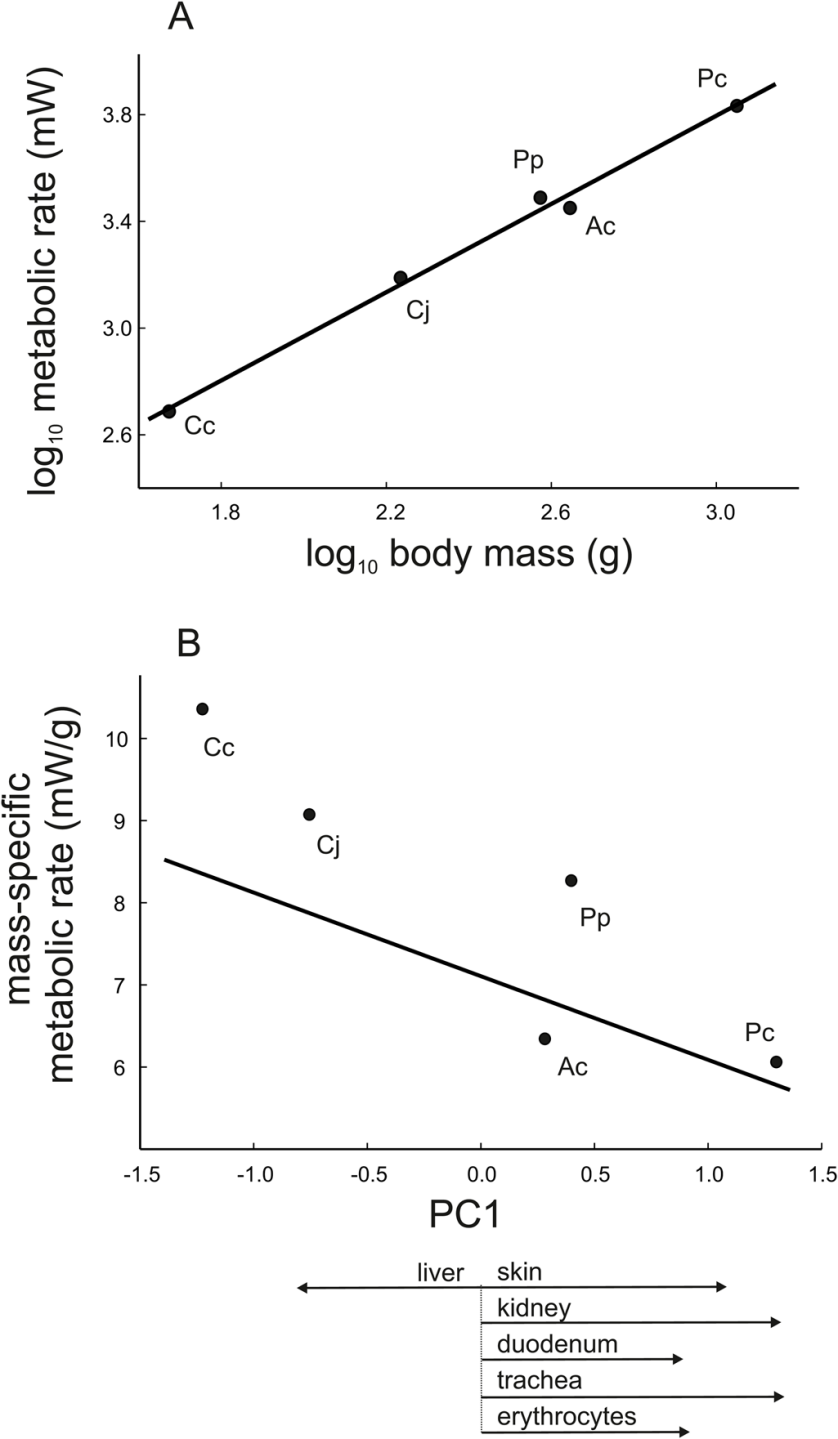
**In birds, metabolic rate increased allometrically with body mass (A) and birds with smaller hepatocytes and larger cells in the other five tissues sampled had lower mass-specific metabolic rates (B).** Lines represent the standardized major axis (A: y=1.32+0.827x; B: y=7.11 −1.018x). Symbols are species means calculated from data for five individuals and are marked with the initials for each species name: Ac, *Alectoris chukar*; Cc, *Coturnix chinensis*; Cj, *Coturnix japonica*; Pc, *Phasianus colchicus*; Pp, *Perdix perdix*. PC1 is the first principal component in the principal component analysis of cell size. Scores for PC1 were used to integrate information on the coordinated changes in cell size. Arrows indicate loading values for cell size in different organs/tissues from PC1 (see Table 1), demonstrating the nature of cell size relationships.

In birds, BMR increased with body mass (r=0.996, *P*=0.0003; Fig. 3A). The mass-scaling exponent was 0.827, indicating a negative allometric relationship (0.703 and 0.973 were the lower and upper limits estimated as 95% confidence intervals). Mass-specific metabolic rates of birds were negatively related to PC1 scores (r=−0.90, *P*=0.036; Fig. 3B). Thus, larger birds that evolved smaller hepatocytes and larger erythrocytes, chondrocytes, enterocytes, skin cells and kidney cells were characterized by lower mass-specific metabolic rates. Note that a low residual variance in the relation of BMR and PC1 to body mass makes it difficult to explore the metabolic effects of PC1 independent of body mass. PC2 scores were not related to mass-specific metabolic rates (r=0.16, *P*=0.80).

## DISCUSSION

In Galliformes birds and Rodentia mammals, species with a larger body mass are consistently characterized by larger cells for five cell types (erythrocytes, enterocytes, chondrocytes, skin epithelial cells, kidney proximal tubule cells) and by smaller hepatocytes. This pattern calls attention to four important phenomena, which have often been missed by earlier studies but can help us gain a better understanding of the nature of the evolutionary processes that drive the origin of differences in traits between species.

The first phenomenon we found demonstrates that species have evolved cells with different sizes, and this evolutionary change shows an organism-wide distribution, indicating that the cellular architecture of tissues has evolved in a coordinated manner throughout the entire body rather than occurring only in individual organs. Previously, coordinated changes in the sizes of different types of cells have rarely been studied, but they have been found, either as part of an evolutionary or phenotypically plastic process, in flies (Azevedo et al., 2002; Czarnoleski et al., 2016; Heinrich et al., 2011; Stevenson et al., 1995), reptiles (Czarnoleski et al., 2017), amphibians, birds, mammals (Kozłowski et al., 2010; Maciak et al., 2014) and plants (Brodribb et al., 2013). Altogether, this emerging evidence suggests that an organism-wide coordination of changes in cell size might be an evolutionarily conserved property of organisms. At the molecular level, the evolution of coordinated changes in cell size is likely to involve alterations in the signaling pathways that control and synchronize cellular growth and proliferation rates in different tissues during development, most likely the TOR (target of rapamycin) and insulin regulatory pathways (De Virgilio and Loewith, 2006; Grewal, 2009; Montagne et al., 1999). Alternatively, but not mutually exclusive of the role of TOR/insulin signaling, organism-wide changes in cell size can evolve through alterations in genome size, e.g. via polyploidization or indel (insertion-deletion) processes (Cavalier-Smith, 2005). It is hypothesized that cytological mechanisms regulate the volume of a cell based on the volume of its nucleus. Given that the volume of a nucleus limits the maximum amount of DNA in a cell, a change in genome size should elicit a change in the size of cell nuclei, which should ultimately correspond to a change in cell size (Elliott and Gregory, 2015; Gregory, 2002; Kozłowski et al., 2003). We are not able to specifically determine which of the two mechanisms played a more important role in the cellular evolution of the birds and mammals we studied, but the effects of genome size alone seem insufficient to explain this evolutionary change. In birds, we found evidence contrary to the idea that the size of cells evolved in tight association with the size of nuclei. The karyoplasmic ratio of erythrocytes differed between species, and the size of erythrocytes and their nuclei were not associated with each other across species, although we acknowledge that the small number of studied species might decrease our statistical power for detecting such an association. Nevertheless, these interspecific patterns do not adhere to common cytological assumptions about the tight association between the sizes of cells and their nuclei and the invariance of karyoplasmic ratios in nature (Cavalier-Smith, 2005). When we compared species of birds or mammals for which we obtained information on genome size, we did not find any evidence that larger cells or cell nuclei were associated with larger genomes. However, these negative results should be treated with caution because only a subset of the studied species had available data on genome size. Additionally, the published data on genome size were obtained from different individuals than the ones we studied, and cell size and genome size are known to vary not only among but also within species. However, not finding statistical connections between cell size and the size of genomes or cell nuclei might also be revealing – it is likely that some species evolved cell size differences through alterations in genome size, whereas in other species, the evolution of cell size involved changes in the properties of the TOR/insulin pathways without a change in genome size. We speculate that if decreases and increases in genome size do not have equal consequences (e.g. deletions result in a higher risk of gene loss than insertions or genome multiplications; the number of gene copies affects the biochemical function of cells), then the evolution of cell size in one direction would proceed mostly through changes in genome size, but changes in the other direction would occur through alterations in TOR/insulin signaling.

Our second finding indicates that changes in cell size in different species evolved in tight connection with changes in body mass; a change in cell size was a part of the mechanism involved in the evolution of adult mass. The role of changes in cell size in either evolutionary or phenotypically plastic changes in body size has been suggested by earlier studies (Adrian et al., 2016; Czarnoleski et al., 2013, 2015b; Hessen et al., 2013; Starostová et al., 2005; Partridge et al., 1999), but rarely has this role been demonstrated simultaneously with reference to information about different cell types (but see Kozłowski et al., 2010; Stevenson et al., 1995). Unlike eutelic organisms, which have constant cell numbers, such as rotifers, nematodes and springtails (Van Voorhies, 1996; Walczyńska et al., 2015), developmental constraints linking the growth of a body with the growth of cells cannot explain the concerted evolutionary changes in cell size and body mass in non-eutelic vertebrates. The results of an artificial selection study on mice demonstrated that cell size in different tissues has evolved independently of body mass (Maciak et al., 2014), indicating that cell size and body mass in non-eutelic organisms have the freedom to evolve independently.

According to our third finding, cell size and body size evolved in concert in a similar manner in birds and mammals despite the independent evolutionary histories of both groups. We view this evolutionary convergence as an indication that concerted evolution between cell size and body mass is not neutral, demonstrating the effects of natural selection rather than random changes. Supporting this adaptive view, *Drosophila melanogaster* Meigen, 1830 evolved similar latitudinal clines in cell size, body size, and the characteristics of their TOR/insulin pathways on the Australian and North American continents despite the independent origins of the two clines (De Jong and Bochdanovits, 2003; Fabian et al., 2012; Paaby et al., 2010). Questions remain regarding this topic. For example, what are the selective advantages and disadvantages of a given cell size, and why have larger species evolved larger cells? Based on information about the BMRs of the studied birds, we found that larger species, which have evolved larger cells (at least in five tissues), have simultaneously evolved lower mass-specific BMRs. A similar pattern in interspecific differences in body mass, cell size and standard metabolic rates has also been found in ectothermic animals, e.g. Madagascar geckos (Starostová et al., 2009). Additionally, large-celled triploids have lower mass-specific metabolic rates than do small-celled diploids in *Cobitis* fish (Maciak et al., 2011) and *Pelophylax* frogs (Hermaniuk et al., 2017). The evidence for a negative association between cell size and mass-specific metabolic rate agrees with the prediction of the TOCS that a body built from larger cells has a relatively lower amount of cell membranes and, therefore, wastes relatively less energy on maintaining operational cell membranes, i.e. in a desired physical and electrochemical state (Czarnoleski et al., 2015a; Kozłowski et al., 2003; Szarski, 1983). Saving on maintenance costs by increasing cell size would be advantageous for organisms that face supply limitation. However, large cells can impair physiological activity by decreasing the total exchange area of cell membranes and decreasing the diffusion efficiency within cells, but such disadvantages of large cells should be of a lesser importance for supply-limited organisms. This hypothesis of the TOCS predicts that large-celled organisms have decreased physiological efficiency, especially when they are challenged by an increased metabolic demand, e.g. caused by increases in physical (catabolic) or biosynthetic (anabolic) work. In support of this hypothesis, a comparative study of the rotifer *Keratella cochlearis* (Gosse, 1851) in different lakes and along a gradient of water depths revealed that larger rotifers that consisted of larger cells occupied cool and oxygenated waters (Czarnoleski et al., 2015b). Additionally, an experimental study of the rotifer *Lecane inermis* (Bryce, 1892) showed that larger rotifers have an advantage in fertility over smaller rotifers in cold and oxygenated waters but that small rotifers that consisted of smaller cells had superior fertility in warm and oxygen-deficient conditions (Walczyńska et al., 2015). To understand the evolution of larger cells in larger species, future studies should investigate whether and why supply limitations increase with body mass and should be based on a wide range of body masses. An intriguing possibility is that larger species become supply limited because they are selected against overinvesting in the network of distribution pathways, which deliver oxygen and nutrients to cells and collect metabolites from cells. To overcome this limitation, vertebrates would need to disproportionally increase the volumes of their main arteries and the total amount of blood relative to their body mass, which would physically handicap larger organisms. It is not surprising that the total volume of blood in a body scales proportionally with body mass, and consequently, less capillary blood on average perfuses a given tissue volume in larger organisms (Dawson, 2003, 2005).

According to our fourth finding, hepatocytes have undergone an evolutionary change in size in the opposite direction than have the other cell types, and this pattern was consistently found in the studied birds and mammals. Earlier, Kozłowski et al. (2010) found a similar pattern in a diverse group of mammalian species but not in amphibians and birds. Interestingly, Czarnoleski et al. (2016) studied cell size differences between two subspecies of the land snail *Cornu aspersum* (O. F. Müller, 1774) and found that the size of cells in their hepatopancreas, the analog of a liver in vertebrates, followed a different pattern than did the sizes of other cell types. Maciak et al. (2014) postulated that the size of cells in a tissue can be functionally associated with supracellular functions and the catabolic versus anabolic activity of a tissue. Following the TOCS, we envision that cell size is matched to a balance between the metabolic demand and the supply of resources in a tissue, but this balance changes locally in a body according to the metabolic activity of a tissue and the local characteristics of the supply system. In fact, both the liver in vertebrates and the hepatopancreas in mollusks are characterized by an especially high level of anabolic activity, which is to a large extent directed toward sustaining the function of other tissues in the body. Additionally, the liver appears to be exceptionally well supplied with oxygen and resources: blood reaches the liver via a dual perfusion system that, at least in mammals, receives ∼25% of the cardiac output (Vollmar and Menger, 2009), and hepatocytes are in direct contact with hepatic capillaries (Kerr, 2010). Finally, independently of other cells in a body, hepatocytes can undergo chromosomal multiplications, which alters their size and translational activity (Anatskaya and Vinogradov, 2007, 2010).

Our work provided crucial insight that cell size should be given greater consideration as an organismal property that undergoes adaptive evolutionary changes among species. This macro-evolutionary view is consistent with emerging conclusions from molecular research that cell size control evolved to optimize the metabolic activity of tissue and organs and ultimately to maximize cellular fitness (Miettinen et al., 2017). Although our data suggest that cell size, body mass and metabolic rates can undergo concerted evolutionary changes, to gain a better understanding of these phenomena, studies of the complex causal links among body size, cell size, physiological efficiency and fitness are needed. If cell size is demonstrated to affect maintenance costs and organismal performance, then its concerted evolution with body mass and metabolic rate suggests that the cellular architecture of the body is adjusted along with many other organismal traits to meet the physiological supply and demand of a given strategy. This interpretation adds a new perspective to views on the biological significance of cells.

## MATERIALS AND METHODS

### Animals

We studied five species of Galliformes birds and six species of Rodentia mammals, representing two distantly related orders of endotherms. Each species was represented by five males. The number of studied animals was dictated by the extreme laboriousness of the histological and microscopic procedures and the cell size measurements. The choice of Galliformes and Rodentia was motivated by their independent origins and differentiation into a wide range of species with large differences in adult mass but minimal changes in their general body plans. In this way, we maximized the studied range of body masses and minimized differences in the biology of the studied species. We were also able to address whether concerted evolutionary changes in cell size and body mass occurred in a similar manner independently in these two groups.

All the birds [common pheasant, *Phasianus colchicus* Linnaeus, 1758; chukar partridge, *Alectoris chukar* (J. E. Gray, 1830); grey partridge, *Perdix perdix* (Linnaeus, 1758); Japanese quail, *Coturnix japonica* Temminck and Schlegel, 1849; and king quail, *Coturnix chinensis* (Linnaeus, 1766)] were obtained from the field station in Ptaszkowo of the Ośrodek Hodowli Zwierząt Łownych in Parzęczewo-Cykowo, Poland. The rodents were obtained from different sources in Poland [house mouse, *Mus musculus* Linnaeus, 1758, from the Jagiellonian Center of Experimental Therapeutics in Kraków; Djungarian hamster, *Phodopus sungorus* (Pallas, 1773), from the Department of Animal Physiology of the Nicolaus Copernicus University in Toruń; brown rat, *Rattus norvegicus* (Berkenhout, 1769), from the Department of Pharmacology of the Medical College of the Jagiellonian University in Kraków; bank vole, *Myodes glareolus* (Schreber, 1780), and common vole, *Microtus arvalis* (Pallas, 1778), from the Institute of Environmental Sciences of the Jagiellonian University in Kraków; and coypu, *Myocastor coypus* (Molina, 1782), from the Pniewy animal husbandry facility]. The animals used in this study were euthanized following the procedures of the institutions from which the animals were obtained, which had been approved by their local ethical committees. The birds and coypus were slaughtered as part of commercial meat production. Other rodents were euthanized after laboratory experiments in which they served as control groups. The donation of animal material and all procedures used in this study followed regulations of the Polish Ministry of Science and Higher Education.

### Histological methods and cell size measurements

Prior to dissection, animals were deprived of food for at least 12 h and then weighed to the nearest 0.01 g (small rodents), 0.1 g (birds), or 1 g (rats and coypu). We took blood samples from each animal with heparinized glass capillary tubes (Medlab, Raszyn, Poland) to prepare blood smears. For Galliformes, blood was taken from the brachial vein, and in rodents, from the caudal vein or jugular vein (only coypus). Blood smears were dried and fixed with methanol (Avantor Performance Materials Poland S. A, Gliwice, Poland) and then stained with Gill's III Hematoxylin (Merck, Darmstadt, Germany) and a 1% ethanol solution of Eosin Y (hereafter, 1% Eosin Y; Analab, Warszawa, Poland) for birds or with 1% Eosin Y for mammals.

After removing feathers or shaving the coat, we collected a skin sample from between the scapulae along the dorsum. We dissected out the middle part of the trachea, the central part of the right lobe of the liver, the descending part of the duodenum and the whole right kidney. The tissue samples were fixed in a 10% buffered solution of formaldehyde (Bio Optica, Milano, Italy). Then, they were dehydrated in ethanol (Linegal Chemicals, Warszawa, Poland), cleared in ST Ultra (Leica, Wetzlar, Germany) and embedded in Paraplast Plus (Leica). Serial sections (4 µm thick) were cut with a motorized rotary microtome (Hyrax M55; Zeiss, Oberkochen, Germany). Slides were stained with Gill's III Hematoxylin and 1% Eosin Y and mounted with CV Ultra (Leica).

Erythrocytes in blood smears, tracheal chondrocytes, hepatocytes and duodenal enterocytes were photographed at a resolution of 0.033 µm per pixel under a light microscope (Eclipse 80i; Nikon, Tokyo, Japan) equipped with a camera (Digital Sight, Nikon) and Lucia Measurement image acquisition software (Lim Laboratory Imaging, Praha, Czech Republic) using a 100×-magnification oil immersion objective. Cells from kidney proximal tubules and epithelial skin cells were photographed at a resolution of 1 µm per pixel using a 40×-magnification objective on an automatized light microscope (BX 51 VS; Olympus, Tokyo, Japan) equipped with a digital camera (XC10, Olympus) and dotSlide (Olympus) image acquisition software. The use of two microscopic systems, including one that was automated, helped to expedite the digitization of microscopic slides and did not bias our results because we consistently used the same system to analyze a given tissue type in all animals.

We used image analysis software to measure cells: ImageJ from the National Institutes of Health (USA) for JPEG images from the Nikon camera and cellSens from Olympus for a specialized image format obtained from the Olympus camera. We outlined 60 randomly chosen erythrocytes per animal and calculated their areas (µm^2^), which was our measure of erythrocyte size. For birds, we used the same method to measure the areas of erythrocyte nuclei. We outlined randomly chosen lacunae in chondrocytes and calculated their areas (µm^2^), which was used as a measure of chondrocyte size. If chondrocytes occurred in isogenic groups, we measured one chondrocyte per group. Cell borders in the remaining tissues were often not clearly visible. Following the methods developed by Wieczorek et al. (2015) and Czarnoleski et al. (2016, 2017), we measured the areas of cell groups in tissue samples from the liver, duodenum and kidney (µm^2^) and the lengths of longitudinal transects of cell groups in skin (µm) samples. After counting the nuclei within the measured areas or along each transect, we calculated the average cell size by dividing the area or transect length by the number of nuclei. To outline areas for measurement, demarcation lines between hepatocytes in liver samples were drawn equidistance between neighboring nuclei. In duodenum samples, we considered only enterocytes in the epithelial mucous membrane in the middle part of villi. We used the basement membrane and the apical surfaces of cells as the lower and upper borders of layers, respectively, and two cell nuclei at two ends of the layer as the beginning and the end of the layer. In kidney samples, we outlined the cross-sectional areas of proximal tubules (without the lumen). In skin samples, we considered epithelial cells of the basal layer, which formed longitudinal transects. The ends of a transect were defined by two nuclei, one at each end of the linear transect of nuclei. We measured the following number of cells per individual ‒ birds: 52-71 in trachea, 88-274 in livers, 46-257 in duodenums, 332-521 in kidneys and 2-102 in skin; mammals: 37-89 in trachea, 70-238 in livers, 37-295 in duodenums, 9-600 in kidneys, 13-166 in skin. Finally, we calculated the average size of each cell type (and the mean size of nuclei in bird erythrocytes) for each animal. Additionally, we calculated mean karyoplasmic ratios for erythrocytes in each bird.

Information about C-values, where C-value is the amount of DNA in a haploid cell (pg), in the studied species was obtained from an online database (Gregory, 2017). If more than one estimate of the C-value was available for a species, we calculated the mean C-value. We found data on C-value in two species of birds and four species of mammals. Therefore, the data for birds were used only for descriptive purposes, whereas the data for mammals were analyzed statistically.

### Respirometry in birds

We measured oxygen consumption rates (cm^3^ O_2_/h) with a paramagnetic analyzer and carbon dioxide production rates (cm^3^ CO_2_/h) using the non-dispersive infrared analysis method (MAGNOS 6G and URAS 10E, respectively, Hartmann and Braun, ABB Group, Zürich, Switzerland). Before passing through the analyzers, which were connected to a personal computer, incurrent air was passed through a column of anhydrous calcium chloride (CaCl_2_; CHEMPUR, Piekary Śląskie, Poland). Birds that underwent metabolic measurements were transferred indoors from the breeding room (king quail and Japanese quail) or from the open aviary (grey partridge, chukar partridge, and common pheasant) and were acclimated to the measurement conditions for 1 h. Birds were deprived of food during the night before the measurements and were weighed prior to respirometry. The birds were placed in plastic chambers with volumes closely matched to the size of each bird (1.2-25.0 l). Chambers with birds were placed in a thermally insulated chamber with the ambient temperature controlled to the nearest 0.1°C (Elmetron PT-217 digital thermometer, Zabrze, Poland). Airflow through the metabolic chamber was stabilized with a mass flowmeter (β-ERG, Warszawa, Poland) at 700-3000 ml/min, depending on the species. The resting metabolic rate at thermoneutrality, representing the BMR, was defined as the lowest 10 min average metabolic rate of each bird. Ambient temperatures matching species-specific thermoneutral zones were known from earlier studies and were as follows: the temperature was maintained at 35.5°C for king quail (Pis and Luśnia, 2005), at 30.5°C for Japanese quail (T.P., unpublished), at 25.0°C for grey partridge and chukar partridge (Pis, 2003, 2010), and at 21.0°C for common pheasant (Górecki and Nowak, 1990). Following Kleiber (1961), we used our measured respiratory quotient values to express metabolic rates in mW. The following energy equivalents of 1 cm^3^ of O_2_ were adopted: 20.27 J for king quail, 19.95 J for Japanese quail, 20.08 J for grey partridge, 20.20 J for chukar partridge, and 20.01 J for common pheasant.

### Statistical analysis

We used the R Statistical Package for the statistical analyses (R Development Core Team, 2011). The number of studied species did not allow us to achieve a satisfactory power for phylogenetically informed analyses (Garland et al., 2005). The analysis of the karyoplasmic ratios of erythrocytes was performed on data from individual birds; other analyses were performed on species means, which were calculated from data for individual animals. To integrate information on cell sizes in different tissue, we performed a PCA on our cell size data, separately for birds and mammals. Scores for the most significant PCs were further used as our integrated measures of cell sizes in different tissues.

The nature of PC loadings was used to explore hypothesis I, which predicted that the evolution of differences in cell size among species involved coordinated changes in the sizes of different cell types. Furthermore, we examined whether this evolution involved changes in genome size/nucleus size. Using information on bird erythrocytes, we analyzed the correlation between the size of erythrocytes and their nuclei using a general linear model (GLM) to compare karyoplasmic ratios for erythrocytes among species. We also analyzed the correlation between genome size (C-value) and cell size (the PC scores). To test hypothesis II about the involvement of changes in cell size in the evolution of body size, we examined the correlation between PC scores and body mass (log_10_-transformed). Analyses were performed separately for birds and mammals. To explore hypothesis III, which predicts that large-celled species have evolved lower mass-specific metabolic rates, we examined the correlation between the mass-specific BMRs of birds and their PC scores. Note that this analysis largely explores the integrated effects of cell size and body mass if, as predicted by hypothesis II, cell size and body mass have evolved in concert. The highly concerted evolution of cell size and body mass makes it impossible to reliably assess the independent effects of cell size and body mass on metabolic rate. Finally, we used a SMATR procedure (Warton et al., 2006) to fit a standardized major axis (SMA) to the log-transformed data for BMR and body mass. Assuming a power relationship between BMR and body mass, we used this to estimate the mass-scaling of BMR. For consistency, we also fitted SMAs for the relationships between PC score and log_10_ body mass and between mass-specific BMR and PC score.

## Supplementary Material

Supplementary information
