# Supplementary material for: Concerted evolution of body mass and cell size: similar patterns among species of birds (Galliformes) and mammals (Rodentia)
Source: Biol Open. 2018 Mar 14;7(4):bio029603. doi: 10.1242/bio.029603 (PMC5936057; doi:10.1242/bio.029603)
Supplement: Supplementary information [file biolopen-7-029603-s1.pdf]

Supplementary information

Table 1S. Raw data on body mass, basal metabolic rate and the sizes of six cell types in Galliformes birds (5 species) and Rodentia mammals (6 species). The data are shown as the mean values calculated for either individual animals or for species. The data on genome size (C-value) were extracted from Gregory (2017). Animal Genome Size Database: http://www.genomesize.com.

Click here to Download Table S1

Biology Open • Supplementary information

Table 2S. Interspecific correlations between body mass (log_{10}) and the mean cell size of six cell types in birds (5 species) and mammals (6 species).

Cell type	Organ/tissue	Measurement unit	Pearson correlation coefficient: birds	Pearson correlation coefficient: mammals
Erythrocytes	Blood	μm^2	0.66	0.97
Enterocytes	Duodenum	μm^2	0.68	0.68
Proximal tubule cells	Kidney	μm^2	0.89	0.73
Epithelial cells	Skin	μm	0.56	0.86
Chondrocytes	Trachea	μm^2	0.89	0.60
Hepatocytes	Liver	μm^2	-0.41	-0.41
